# R430: A potent inhibitor of DNA and RNA viruses

**DOI:** 10.1038/s41598-018-33904-y

**Published:** 2018-11-09

**Authors:** Leonardo D’Aiuto, James McNulty, Caroll Hartline, Matthew Demers, Raj Kalkeri, Joel Wood, Lora McClain, Ansuman Chattopadhyay, Yun Zhi, Jennifer Naciri, Adam Smith, Robert Yolken, Kodavali Chowdari, Carlos Zepeda-Velazquez, Chanti Babu Dokuburra, Ernesto Marques, Roger Ptak, Paul Kinchington, Simon Watkins, Mark Prichard, David Bloom, Vishwajit Nimgaonkar

**Affiliations:** 1 Department of Psychiatry, Western Psychiatric Institute and Clinic, University of Pittsburgh School of Medicine, 3811 O’Hara Street, Pittsburgh, PA 15213 USA; 2 Department of Chemistry and Chemical-Biology, McMaster University, 1280 Main Street West, Hamilton, Ontario, L8S 4M1 Canada; 3 University of Alabama at Birmingham, UAB School of Medicine, 1720 2nd Ave. S., Birmingham, AL 35294-3412 USA; 4 Department of Infectious Disease Research, Drug Development, Southern Research Institute, 431 Aviation Way, Frederick, Maryland 21701 USA; 5 Magee-Women’s Research Institute, 204 Craft Ave, Pittsburgh, PA 15213 USA; 6 Department of Pharmacology and Pharmaceutical Sciences, School of Medicine, Tsinghua University, Beijing, China; 7 Division of Neurovirology, Department of Pediatrics, Johns Hopkins University School of Medicine, 600 North Wolfe Street, Blalock 1105, Baltimore, MD 21287 USA; 8 University of Pittsburgh, Department of Infectious Diseases and Microbiology, 9022 BST3, 3501 Fifth Avenue, Pittsburgh, PA 15260 USA; 9 Department of Ophthalmology, University of Pittsburgh School of Medicine, 1016 Eye and Ear Institute, Pittsburgh, PA 15213 USA; 10 Department of Cell Biology, University of Pittsburgh, 3500 Terrace Street, S362 Biomedical Science Tower (South), Pittsburgh, PA 15261 USA; 11 Department of Molecular Genetics & Microbiology, University of Florida College of Medicine, Box 100266 JHMHC, Gainesville, FL 32610-0266 USA; 12 Department of Human Genetics, Graduate School of Public Health, University of Pittsburgh, Pittsburgh, PA 15213 USA; 13 Molecular Biology Information Service, Health Sciences Library System, 3550 Terrace Street, Pittsburgh, PA 15261 USA

## Abstract

Acyclovir (ACV) is an effective antiviral agent for treating lytic Herpes Simplex virus, type 1 (HSV-1) infections, and it has dramatically reduced the mortality rate of herpes simplex encephalitis. However, HSV-1 resistance to ACV and its derivatives is being increasingly documented, particularly among immunocompromised individuals. The burgeoning drug resistance compels the search for a new generation of more efficacious anti-herpetic drugs. We have previously shown that trans-dihydrolycoricidine (R430), a lycorane-type alkaloid derivative, effectively inhibits HSV-1 infections in cultured cells. We now report that R430 also inhibits ACV-resistant HSV-1 strains, accompanied by global inhibition of viral gene transcription and enrichment of H3K27me3 methylation on viral gene promoters. Furthermore, we demonstrate that R430 prevents HSV-1 reactivation from latency in an *ex vivo* rodent model. Finally, among a panel of DNA viruses and RNA viruses, R430 inhibited Zika virus with high therapeutic index. Its therapeutic index is comparable to standard antiviral drugs, though it has greater toxicity in non-neuronal cells than in neuronal cells. Synthesis of additional derivatives could enable more efficacious antivirals and the identification of active pharmacophores.

## Introduction

Human herpes viruses (HHV) infect more than 3.7 billion people world-wide^1,2^, causing substantial morbidity^3,4^. Herpes Simplex virus, type 1 (HSV-1), a prototypic HHV causes mucosal infection, encephalitis and is a leading cause of blindness in the USA^1^. Following primary mucosal infection, virions migrate to sensory ganglia where they establish a latent state characterized by the reversible retention of non-replicating, episomal viral genomes^5–7^. Therapeutic options for HSV-1 infections are limited to treating recurrences with nucleoside analogues like Acyclovir (ACV) and it has not been possible to develop effective vaccines^8^. ACV and its analogues can abort and suppress lytic infection with a high margin of safety. It can be administered to pregnant individuals. Its common side effects are restricted to nausea and vomiting. It is a potent nucleoside inhibitor, with antiviral effects in the micromolar range. Though ACV is potent and safe, resistance to ACV has been reported, with incidence rates up to 7.1% in immunocompromised persons^9–13^. Unlike HSV-1, effective and safe antiviral drugs are currently unavailable for other HHVs. A continued search for new drugs against HHVs is thus an urgent public health necessity. Agents that are effective against additional herpes viruses are desirable, because an individual can be infected with more than one HHV during her lifetime^14^.

Extracts of plants belonging to the *Amaryllidaceae* family have extensive antiviral activities against DNA and RNA viruses^15^. Renard-Nozaki *et al*. first reported that synthetic *Amaryllidaceae* alkaloids inhibited replication of HSV-1 in monkey epithelial (Vero) cells^16^. Subsequently, Gabrielsen *et al*. reported on a series of 23 Amaryllidaceae isoquinoline alkaloids and synthetic analogues that possessed inhibitory effects against several viruses, albeit with relatively low margins of safety, against selected flaviruses (Japanese encephalitis, yellow fever, and dengue viruses) as well as bunyaviruses (Punta Toro, and Rift Valley fever viruses)^17^. Pancratistatin, another *Amaryllidaceae* derivative with antineoplastic properties, and its 7-deoxy analogue increased survival in a Japanese-encephalitis-virus-infected mouse model^17^. The relatively low margins of safety spurred us to screen ten other Amaryllidacea alkaloid derivatives^18^. R430 (3-*epi-trans*-dihydrolycoricidine) inhibited productive infection with the HSV-1 KOS strain effectively in both monkey epithelial (Vero) cells and in neuronal cells derived from human induced pluripotent stem cells (hiPSC-neurons), with an estimated therapeutic index (TI) over 500^18^. It also significantly reduced HSV-1 reactivation from latency in our hiPSC-N based model^18,19^, suggesting effects on the initiation of productive infection from the latent state. R430 caused substantial reduction in the expression of not only viral DNA polymerase, but also the viral IE gene ICP4. R430 also inhibited varicella zoster virus (VZV) lytic infection more potently than ACV^18^. A hydroxy analogue of R430 was recently reported to have potent activity to the Zika virus (ZIKV)^20^, suggesting that R430 and its derivatives could inhibit a spectrum of DNA and RNA viruses.

In the present study, we pursued three lines of enquiry. First, we investigated the potency of R430 against additional HSV-1 strains, including two ACV-resistant HSV-1 strains. We also analyzed the ability of R430 to prevent HSV-1 reactivation from latency in an *ex vivo* rodent model. Second, aspects of the mechanism/s of action of R430 were studied through its inhibitory effect on the expression of HSV-1 genes in hiPSC-derived neurons and its effects on HSV-1 chromatin in infected hiPSC-N. Finally, we investigated the range of antiviral effects of R430 by estimating its potency and toxicity in cellular infection models of Zika virus (ZIKV), Herpes Simplex virus, type 2 (HSV-2), human cytomegalovirus (hCMV), murine CMV (mCMV), Hepatitis B virus (HBV) and Hepatitis C virus (HCV).

## Results

### R430 efficiently inhibits ACV resistant HSV-1 strains

**Figure 1:**
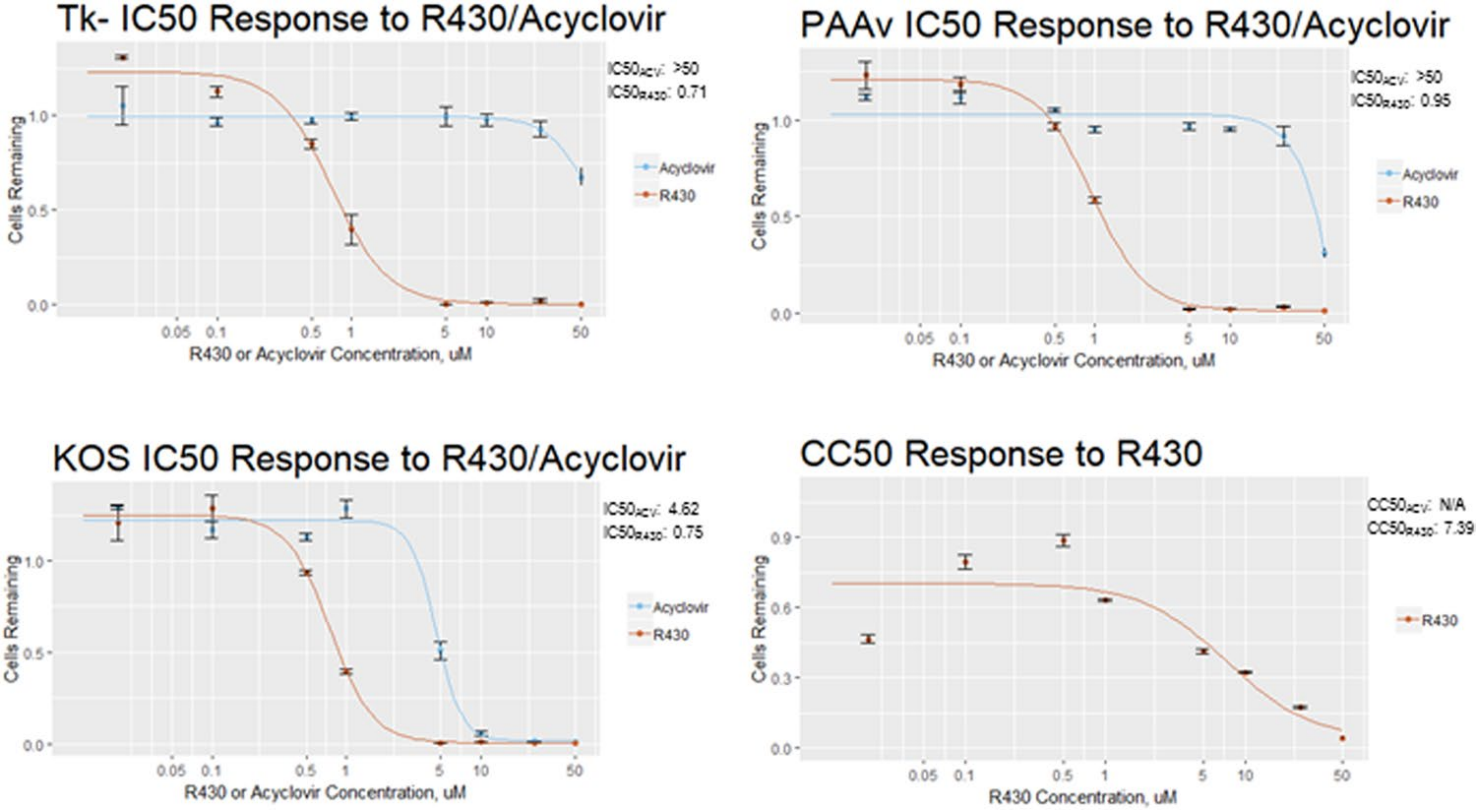
R430 is effective against acyclovir-resistant strains of HSV-1. Vero cells were infected with ACV-resistant HSV-1 strains Tk- and PAAv, or HSV-1 KOS strain, and incubated with acyclovir or R430 at concentrations varying from 0.1–50 µM. At 48 hours post-infection cells were fixed and stained for ICP4 protein, counterstained with Hoechst 33342, and imaged with Nikon AS1 microscope. Cells were counted and IC50 was determined using the drc package for R, based on the proportion of positive-staining cells. CC50 for R430 was determined using drc package based on total number of remaining cells. No CC50 is reported for ACV due to negligible toxicity.

R430 is more potent than ACV against the HSV-1 KOS strain^18^, but its potency against other strains is unknown. Therefore, R430 and ACV were compared against the *tk-* strain of HSV-1 that lacks thymidine kinase activity^21^ and the PAAv strain that has developed mutations in viral DNA polymerase following incubation with phosphonoacetic acid^22^. Both strains have been reported to be resistant to ACV. These experiments were conducted in hiPSC-derived neural progenitor cells (NPCs) that are sensitive to HSV-1 infection^23^. R430 showed higher potency than ACV against both strains, though ACV caused less cytotoxicity (Fig. 1).

### R430 prevents reactivation of HSV-1 from latently infected explanted murine trigeminal ganglia

Our prior studies of R430 indicated inhibition of HSV-1 reactivation in a novel hiPSC-based model of latent infection. We therefore investigated its effects on an established mouse explant co-cultivation model^24^, wherein axotomy and excision of trigeminal ganglia (TG) from latently infected mice results in reactivation events *in vitro*. To estimate the effects of R430 in inhibiting HSV-1 reactivation from latency, TG of 39 HSV-1 latently infected mice were explanted, and the 78 TG were excised and co-cultured with rabbit skin cells (RSC) for reactivation, with daily examination of mouse explanted whole TG co-cultures. By 21 days post-explant, 31/39 of the vehicle treated TG showed evidence of reactivation (cytopathic effect and spreading foci of infection), whereas none (0/39) of the R430 treated TG showed a reactivation event (chi square = 51.45, p = 2.31 × 10–14, Fisher’s exact test). Since R430 would be predicted to potentially block replication of the virus on the indicator cells, we confirmed these results by subjecting supernatants from both vehicle- and R430-treated TGs to PCR for HSV-1 DNA. As expected, the R430 mediated inhibition of reactivation could be attributed to inhibition of the HSV-1 induced replication in the RSC cells, since none of the R430 treated supernatants contained detectable viral DNA, whereas the vehicle treated supernatants did.

### R430 substantially reduces transcription of HSV-1 genes in infected hiPSC-neurons

**Figure 2:**
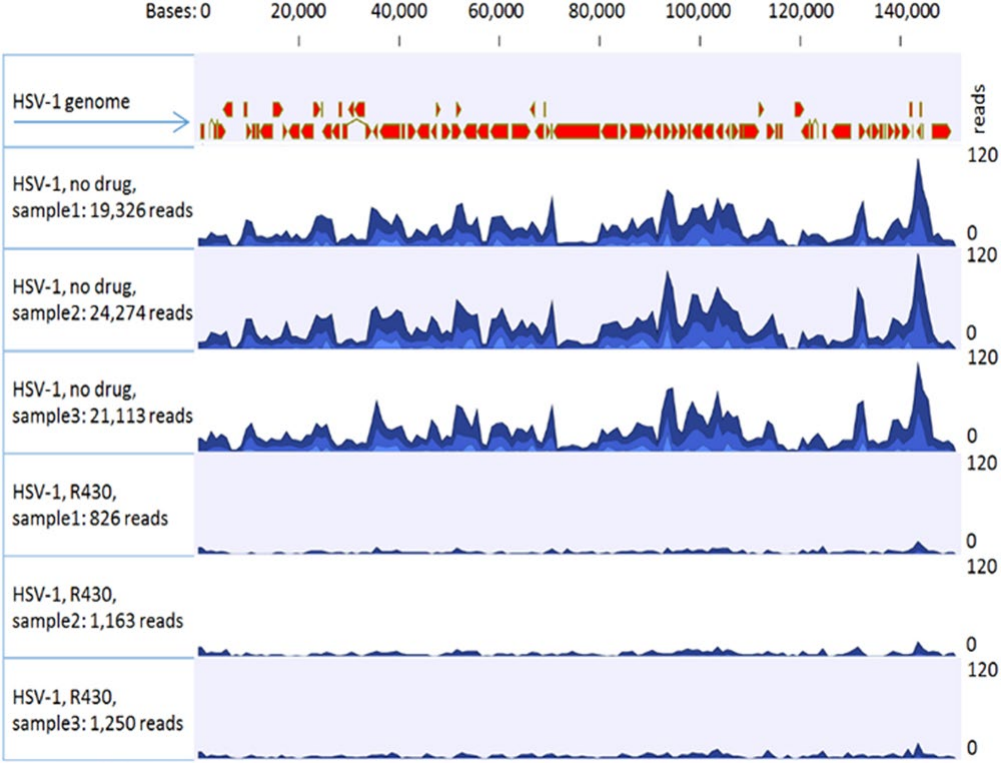
Effects of R430 on HSV-1 transcript levels. hiPSC-neurons were incubated with HSV-1 (MOI 0.3) and R430 (10 µM) or vehicle in triplicate. Total RNA was extracted, quantified and sequenced following ribosome depletion. Sequences were trimmed and mapped to GRCH38 using sequence and annotation provided by Ensembl (release 82). The remaining unmapped reads were collected and mapped to the Human herpesvirus 1 strain KOS genome (GenBank: JQ780693.1).

hiPSC-N were infected with the HSV-1 KOS strain (MOI = 0.3) and treated with R430 (10 µM) or vehicle (DMSO) for 12 hours (see Supplementary Fig. 1). Separately, uninfected cells were incubated with R430 (10 µM) or vehicle for the same duration. Following extraction, viral and host RNA sequences were analyzed. A marked reduction in all viral transcripts was noted in infected cells treated with R430 (HSV-1 infected vs HSV-1 infected + R430, t = −14.11, 4 df, p = 0.00015; Fig. 2, Table 1). These results indicate that R430 blocks the HSV-1 lytic phase, beginning with the inhibition of the immediate early (IE) genes.

**Table 1:** Effects of R430 on HSV-1 and host gene transcription.

Assay conditions	RIN	Host genome reads	Viral genome reads
Uninfected, no R430	9.43 (0.15)	103.89 × 10^6^ (2.33 × 10^6^)	Not detected
Uninfected, with R430	9.53 (0.06)	100.46 × 10^6^ (8.65 × 10^6^)	Not detected
Infected, without R430	9.60 (0.17)	93.99 × 10^6^ (8.25 × 10^6^)	21,571 (2505.59)
Infected, with R430	9.5 (0.10)	105.82 × 10^6^ (13.39 × 10^6^)	1,079 (223.95)

hiPSC-neurons were incubated with HSV-1 (MOI 0.3) and R430 (10 µM) in triplicate; appropriate control conditions were included. Total RNA was extracted, quantified and sequenced following ribosome depletion. Sequences were trimmed and mapped to GRCH38 using Ensembl (release 82) to estimate human transcript counts. The remaining unmapped reads were collected and mapped to the Human herpesvirus 1 strain KOS genome (GenBank: JQ780693.1) to estimate viral transcript counts. The mean number of human and viral transcripts are shown (standard deviations in brackets). The following comparisons were statistically significant:

Viral transcripts in (uninfected + R430) vs (uninfected + vehicle) conditions: t = −14.11, p = 0.00015; Human transcripts in (uninfected + R430) vs (uninfected + vehicle): t = −0.61, p = 0.573.

Human transcripts in (infected + R430) vs (infected without R430): t = 1.27, p = 0.271.

There were no significant changes in human transcripts in the HSV-1 infected vs uninfected conditions (t = 0.11, p = 0.13). RIN: RNA integrity number.

RIN: RNA integrity number. All values shown as mean (standard deviation) from triplicate experiments. R430 (10 µM) was used where specified.

Viral expression in (uninfected + R430) + vs uninfected: t = −14.11, p = 0.00015.

Effects of R430 on host: (uninfected + R430) vs uninfected: t = −0.61, p = 0.573.

(infected + R430) vs (infected without R430): t = 1.27, p = 0.271.

Effects of virus on host: infected vs uninfected: t = 0.11, p = 0.13.

### R430 alters the expression of a proportion of human genes

A total of 13,175 human genes were expressed at TPM >= 5 in untreated cells in the prior experiment. When hiPSC-N were incubated with R430 or vehicle in the absence of HSV-1, the expression of 1,446/13,175 genes (10.9%) was significantly altered (FDR corrected p < 0.05, maximum group mean expression TPM >= 5). Using quantitative RT-PCR, we confirmed alterations in mRNA levels for 6 selected genes that were significantly altered by R430 in the RNA sequencing analyses (Supplementary Fig. 2).

Using Ingenuity Pathway Analysis (IPA), we identified canonical pathways that were significantly altered when hiPSC-neurons were incubated with R430 or vehicle (Supplementary Fig. 3). Statistical significance was calculated using right-tailed Fisher Exact Probability Tests; biological pathways showing p-value < 0.05 were considered statistically significant. The ten most significantly altered pathways are shown in Supplementary Fig. 3. The eukaryotic initiation factor 2 (EIF2) signaling pathway was most significantly altered and was the only one with imputed down-regulation (z score: −4.802, p-value = 2.83E-18). IPA analyses also indicated nett upregulation in the IL-1, TNFR1 and TNFR2 signaling pathways; the direction of change could not be imputed conclusively in the remaining six pathways that were significantly altered. Notably, R430 increased transcript levels of Janus kinase 2 (JAK2) (FC = 1.42, p = 2.4e-12) and the signal transducer and activator of transcription 3 (STAT3, FC = 159, p = 1.3e-16). Pathway analyses of HSV-1 infected cells treated with R430 or vehicle showed similar patterns of change (data not shown). Similar analyses were not conducted in relation to HSV-1 infection alone, because transcript levels for only one gene were signficantly altered in infected cells.

### Effects of R430 on viral gene promoters methylation

**Figure 3:**
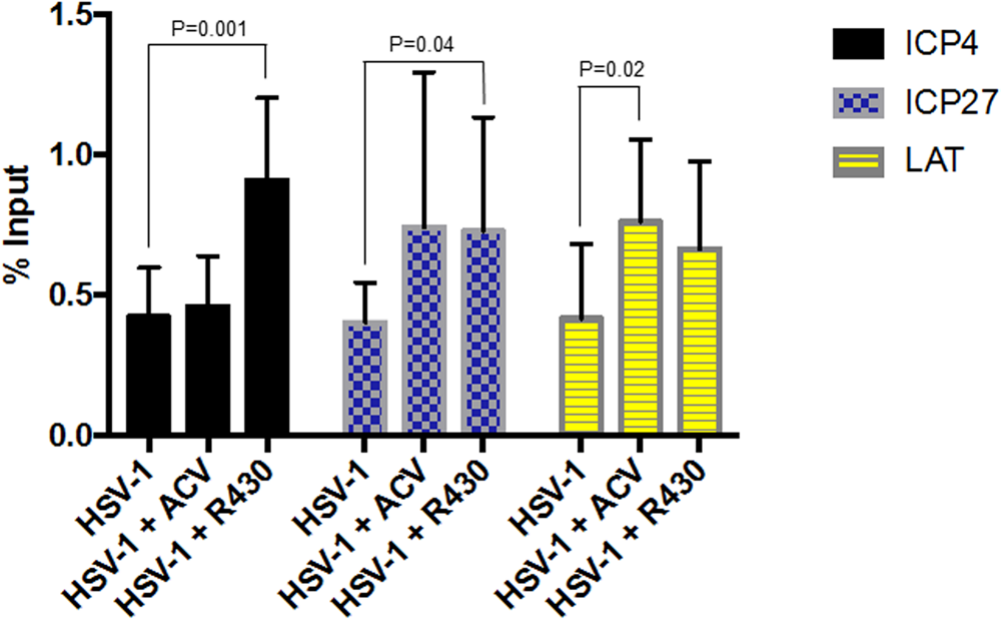
Enrichment of repressive mark H3K27me3 at HSV-1 genes in infected hiPSC-neurons treated with R430 (10 µM) or ACV (50 µM) for 24 hours. ChIPs using anti-histone H#K27me3 antibody were subjected to real-time PCR using primers specific for the HSV-1 target genes indicated, and the results were graphed as % Input. The data represent an average of three independent experiments. Error bars represent standard deviations.

To evaluate patterns of HSV-1 gene inactivation through histone modification, repressive marks with H3K27me3 on the promoters of viral genes ICP4, ICP27 and LAT in infected neuronal cultures exposed to ACV (50 uM) and R430 (10 uM) for 24 hours were assayed using chromatin immunoprecipitation (ChIP). A significant enrichment of H3K27me3 was observed at the promoter regions of ICP4 and ICP27 viral genes in infected cells exposed to R430 and only at the LAT promoter in ACV-treated infected cells (Fig. 3).

### R430 does not induce autophagy

In view of the significant effects of R430 on human gene transcript levels, we explored whether it induced autophagy. When ARPE-19, a human retinal pigment epithelial cell line, was incubated with R430 (10 uM), cells developed a level of rounding, indicating some toxicity, but at 1 uM R430, cells showed no observable phenotypic effects. No puncta formation was observed in LC3 for any concentrations tested, indicating that the antiviral activities are not due to the induction of autophagy and its antiviral consequences on HSV-1 replication.

### Potency and toxicity of R430 against additional RNA and DNA viruses

**Figure 4:**
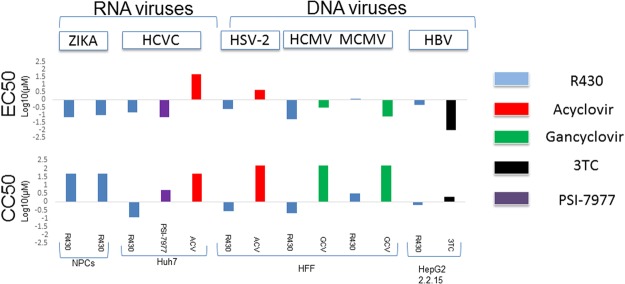
Potency and toxicity of R430 and conventional antivirals on selected RNA and DNA viruses. A panel of antiviral drugs were incubated with cell cultures and R430, conventional antivirals or vehicle as described, in triplicate. The 50% inhibitory concentration (EC50) values are shown (upper panel). Separately, the 50% cytotoxic concentrations (CC50) were estimated following incubation of cell lines with drugs or vehicle in the absence of virus. ZIKA: Zika virus; HCV: Hepatitis C virus, HSV-2: Herpes Simplex virus, type 2; HCMV: Human cytomegalovirus; MCMV: murine cytomegalovirus; HBV: Hepatitis B virus.

The potency of R430 in inhibiting infections with ZIKV, HSV-2, hCMV, mCMV, HBV and HCV are shown in Fig. 4 and Supplementary Table 1. Though the 50% inhibitory concentration (EC50) values for R430 were comparable to the reference drugs against some of the viruses tested, the 50% cytotoxic concentration (CC50) values were relatively lower in non-neuronal cells, leading to lower selectivity index (SI50) values than the reference drugs in these cell lines. Specific antiviral effects were demonstrated with Zika and HSV-1, using a conventional cutoff value of 5 for SI50. The potency of R430 against HSV-2 and HCMV could not be accurately determined as cytotoxicity was observed in the primary cells with extended drug exposure.

**Figure 5:**
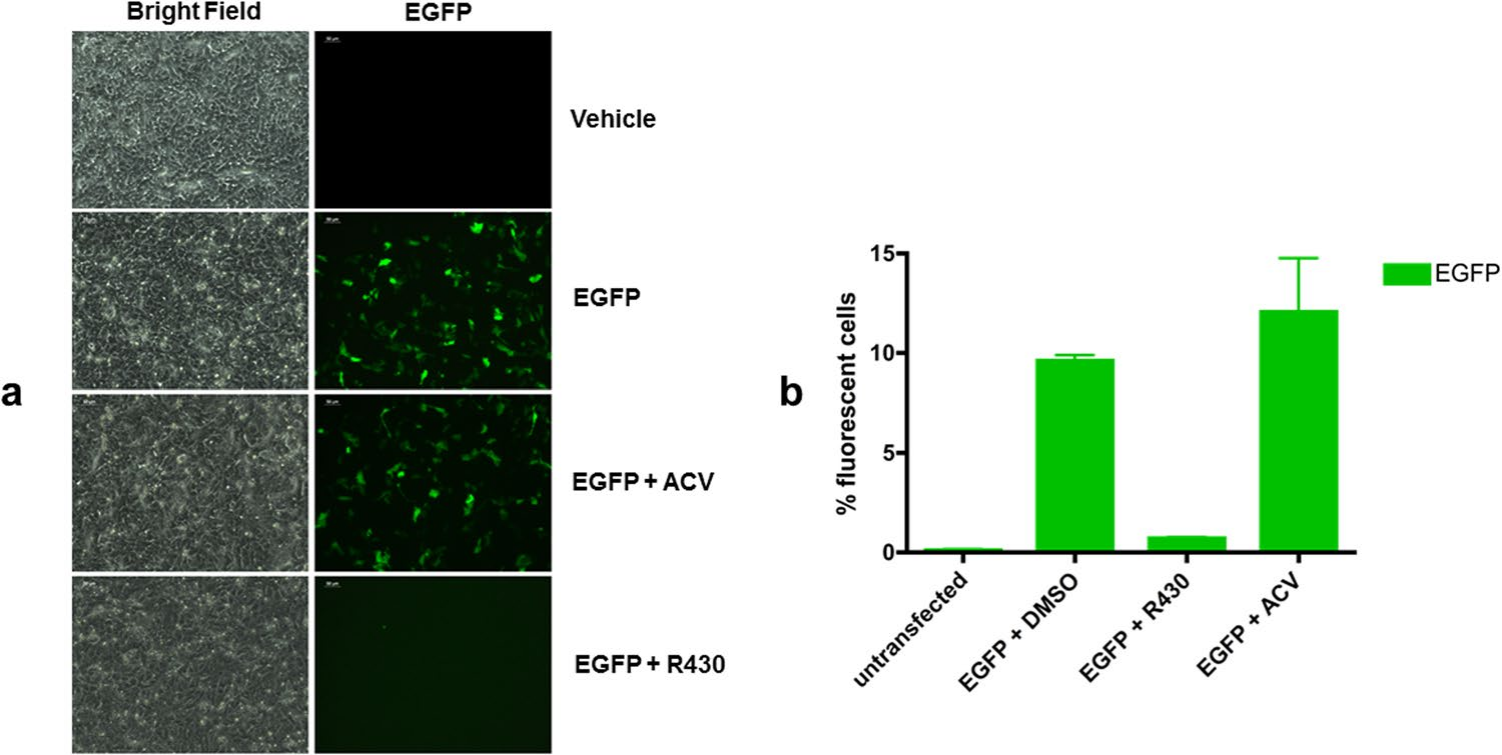
R430 suppresses EGFP expression in a transient plasmid transfection assay. Vero cells were transfected with pEGFP-N1. Two hours after the transfection cells were treated with R430 (10 µM) or ACV (50 µM). The corresponding EGFP expression under different conditions is depicted (**a**). The percentage of EGFP-positive cells was measured 24 hours after the transfection by flow cytometry (**b**).

R430 inhibits the expression of reporter genes in plasmid transfection assays. The ability to effectively inhibit the transcription of HSV-1 IE genes raised the possibility that R430 targets host-specific antiviral mechanisms or it inhibits gene expression from exogenous nucleic acids. To assess this question, we investigated the effect of R430 on the expression of the EGFP reporter gene in a transient plasmid transfection assay. Vero cells were transfected with pEGFP-N1 plasmid. Two hours after the transfection, cells were treated with 10 µM R430 or 50 µM ACV. The proportions of EGFP-positive (EGFP^+^) cells in untreated and treated cultured cells were analyzed 24 hours after the transfection using flow cytometry (FC). R430 treatment caused an approximately 12-fold reduction in the percentage of EGFP^+^ cells (Fig. 5). Conversely, no reduction in the percentage of EGFP^+^ cells was observed in transfected cultures exposed to ACV. Taken together, these results, beside highlighting a difference in the mechanism of action between R430 and ACV, show the ability of R430 to inhibit exogenous gene expression.

As the pEGFP-N1 plasmid incorporates a CMV promoter, we repeated the experiments using pEF-GFP or px458 that are driven by EF1-α and chicken β actin promoters, respectively (see methods). Unlike ACV, R430 inhibited reporter genes in both plasmids (Supplementary Fig. 4).

## Discussion

Following our earlier studies indicating an inhibitory effect of R430 on productive HSV-1 infection in monkey Vero cells and hiPSC-neurons^18^, we show that R430 effectively prevents HSV-1 reactivation in a murine model of HSV-1 latency. The absence of reactivation in mouse TG explants is consistent with our earlier studies in a hiPSC-N model of latent HSV-1 infection^18^. Importantly, R430 also inhibits clinical strains of HSV-1 that are resistant to ACV. These results are highly relevant because the incidence of HSV-1 encephalitis is rising^25,26^ and there is growing concern about ACV resistant strains^12^.

The transcriptome analyses indicate substantial reduction in all HSV-1 transcripts in acutely infected hiPSC-neurons treated with R430. As R430 was added at 2 hpi, these effects are likely independent of effects on viral entry. In particular, the marked reduction of HSV-1 IE gene transcripts suggests that R430 influences the early stages of the HSV-1 lytic phase. These results comport with our prior analyses showing reduced transcription and translation of the viral IE gene ICP4 and HSV-1 DNA polymerase using qPCR and Western assays^18^. The reduction of HSV-1 transcripts is followed by an enrichement of the repressive H3K27me3 marker at the promoter region of ICP4 and ICP27 HSV-1 IE genes in response to 24 hours R430 treatment. The change in the viral chromatin composition in R430-treated may be a consequence of the upregulation of STAT3, which could potentially cause repression of HSV-1 through interaction with the master regulator KAP1, as described for other HHV, such as Kaposi’s Sarcoma-Associated Herpesvirus, and Epstein-Barr virus (EBV)^27,28^. However, the involvment of KAP1 in the silencing of the HSV-1 genes needs to be determined. Unlike ACV, which primarily inhibits viral DNA replication, R430 inhibits early stages of HSV-1 lytic phase. Furthermore, in contrast to ACV, R430 reduced the expression of a reporter gene in transient plasmid transfection assays. Together with its potency against strains resistant to ACV, these results indicate that the mechanisms of antiviral effects of R430 are distinct from ACV.

Our analyses of a panel of viruses were stimulated by our prior report indicating the potency of R430 against VZV^18^, and our recent discovery that OR430, a derivative of R430, is efficacious against ZIKV^20^. Consistent with the earlier reports, we found that R430 exerts antiviral activity against RNA viruses like ZIKV (Brazilian and Cambodian strains). Although treatment with R430 reduced viral production of HCV, HSV-2, CMV, and HBV, the higher cellular toxicity observed in these assays precludes the conclusion that R430 has specific antiviral effects on these viruses. Additional studies, such as using less toxic R430 analogs, would facilitate the investigation. Our data thus support and extend earlier reports suggesting antiviral potency of naturally occurring Amaryllidacea alkaloids against a range of DNA and RNA viruses^16–18^.

The mechanism/s uderlying the inhibitory effects of R430 on DNA and RNA viruses (in partcular, the inhibitory effects on the transcription of HSV-1 lytic genes or Zika replication) are not presently clear. The antiviral effects likely result from a combination of the diverse changes in viral and host gene transcription. In view of the reduced transcription of three transfected plasmids driven by different promoters, a general inhibitory effect on the regulation of transcription is plausible. Its effects on human gene pathways related to innate immunity and translational mechanisms also need to be investigated further. An understanding of these mechanisms will pave the way for additional novel effecacious antiviral drugs. HSV-1 encodes an RNAse that acts early in infection^29,30^, thus the lack of substantial changes in host RNA in infected cells is surprising. The published work was conducted in non-neuronal cells; it is possible that the HSV-1 RNAse activity differs in neuronal cells. This question merits further enquiry since it may highlight difference in activity of HSV-1 in neuronal cells versus non-neuronal cells.

The main drawback of R430 is its variable cellular toxicity, particularly noted in fibroblasts and hepatocytes. On the other hand, R430 had relatively low toxicity in NPCs (this study) and human iPSC derived neuronal cells and ARPE-19 retinal pigment epithelial cells, comparable to ACV^18^. Thus, R430 appears to have lower toxicity in neuronal cells than in non-neuronal cells. The basis for the varied profile of cellular toxicity is uncertain at present and needs to be investigated further.

There are some limitations in our analyses. In hiPSC-neurons, only one host cellular gene showed altered transcription following HSV-1 infection, indicating that the effect of R430 was tested at a relatively early stage of HSV-1 infection. Additional studies at later stages of infection, as well as time course studies and additional pharmacokinetic studies of R430 are required. The ChIP analyses were performed at 24 hpi, when significant enrichment of H3K27me3 was detectable at the ICP4 and ICP27 promoters (*p* = 0.001, and *p* = 0.04, respectively). Further analyses following longer duration of infection would be informative as extended time periods may be needed to establish latency and more replicable results. Studies in which infected cells are exposed to R430 for at least 7 days would be more instructive to evaluate whether R430 indeed induces heterochromatization of the viral genome and consequent viral latency. Additional toxicity studies are also needed in rodent models. We are currently evaluating its therapeutic potential as a topical antiviral agent, e.g., to treat ocular infection caused by HSV-1 – the main cause of viral blindness in the USA.

In conclusion, R430 exhibits a remarkable inhibitory activity against ACV-resistant HSV-1. It prevents HSV-1 reactivation from latency in an *ex vivo* mouse model and displays antiviral effects against other DNA viruses (HSV-2, HCMV, MCMV, HBV) and RNA viruses (Brazilian and Cambodian Zika strains of ZIKV, HCV). These antiviral effects are likely complimented by a profile of favorable effects on host cellular defense mechanisms. On the other hand, R430 shows variable levels of toxicity in non-neuronal cells. Less toxic derivatives of R430 that retain its antiviral properties are therefore needed.

## Methods

### Cell cultures and transfections

#### Cell lines

hiPSC line 73-56010-02 was employed in this study^23,31^. It was generated from fibroblasts derived from skin biopsy samples which were collected from healthy individuals via 4 mm full thickness punch biopsies under local anesthesia. Participants donated biopsies through their participation in the study “Family-Based Genome-Wide Methylation Scan in Neurocognition and Schizophrenia,” in which subjects were enrolled at the University of Pittsburgh (PITT). The University of Pittsburgh’s Institutional Review Board reviewed and approved the study, and all methods were performed in accordance with the guidelines and regulations of the University of Pittsburgh’s Institutional Review Board. Written informed consent was obtained from all participants before participating in any study-related procedures. The hiPSCs were established at the National Institute of Mental Health (NIMH) Center for Collaborative Studies of Mental Disorders-funded Rutgers University Cell and DNA Repository (http://www.rucdr.org/mental-health) (RUCDR). hiPSCs were cultured in mTeSR1 medium (STEMCELL Technologies).

The African green monkey (Vero) cell line (ATCC) was cultured with Dulbecco’s modified eagle medium (DMEM) supplemented with 10% FCS, 100 μg Streptomycin/ml, 100 U Penicillin/ml (all reagents from Chemicon/Millipore). Cultures were maintained in a humidified chamber in a 5% CO_2_/air mixture at 37 °C.

The NPCs and hiPSC-neurons were generated from hiPSCs as previously described^32^.

#### Transfections

DNA transfections into Vero cells were carried out by lipofectamine 2000 (Invitrogen). Cells in exponential growth were seeded (7.5 × 10^4^) into 12-well plates the day before transfection. Cells were transfected with 2 µg of pEGFP-N1. After two hours, transfected cells were exposed to 10 µM R430 or 50 µM ACV. All transient transfections were carried out in triplicate. Twenty-four hours after the transfection, the EGFP expression was analyzed using the flow cytometer. Mock-transfected Vero cells were used as negative controls whereas cells transfected with pEGFP-N1 plasmid served as positive controls.

In separate experiments, R430 inhibition of plasmid expression was tested by transfecting HEK293T cells with one of two plasmids, pER-GFP or px458, using Fugene HD (Promega E2311). At 2 hours post-transfection R430-containing media were added to yield a final concentration of 1, 10 or 50 µM R430, or DMSO control, as well as 50 µM ACV (with four replicates each). At 48 hours post-transfection cells were evaluated for GFP expression by microscopy, then harvested and stained with Viability 780 dye (BioGems 62910-00), fixed with 4% paraformaldehyde and counted on a BD LSR Fortessa FACS instrument. Dead cells and debris were excluded from analysis and percentage of GFP-expressing cells were calculated for each sample.

#### Cellular assays for additional viruses

R430 was tested for antiviral activity against several viruses by reference laboratories namely the University of Alabama (UAB, Birmingham Alabama: Herpes Simplex virus, type 2 (HSV-2), human and murine cytomegalovirus (HCMV and MCMV, respectively), and the Southern Research Institute (SRI, Frederick, Maryland: Hepatitis B (HBV) and Hepatitis C virus (HCV).

#### HSV-2 and CMV

Antiviral activity against HSV-2 G and HCMV AD169 was evaluated using cytopathic effect (CPE) reduction assays by standard methods^33^. Briefly, monolayers of human foreskin fibroblast (HFF) cells were infected in triplicate with each of the viruses listed above at an MOI of approximately 0.001 pfu per cell, along with drugs. At 7 days (d) following infection, CPE was evaluated in cells infected with HSV-2, and at 14 d in cells infected with HCMV. For the Smith strain of MCMV, MB352 cells (ATCC CRL-2821) were seeded into 96-well plates and infected as described above. At 7d following infection, total DNA was harvested with a Wizard kit SV96 genomic DNA (Promega, Madison, WI). MCMV viral DNA was quantified by qPCR with primers 5′-TCA GCC ATC AAC TCT GCT ACC AAC-3′ and 5′-ATC TGA AAC AGC CGT ATA TCA TCT TG-3′ and FAM labeled probe 5′-TTC TCT GTC AGC TAG CCA ATG ATA TCT TCG AGC-3′. For each virus, concurrent cytotoxicity studies were performed on the same cells using the same compound exposure using CellTiter-Glo (Promega), which also quantified the cell counts. The control compounds ACV and ganciclovir (GCV) were purchased from the University of Alabama Hospital Pharmacy. Data obtained were used to calculate concentrations of compounds sufficient to inhibit viral replication by 50% (EC50) and cell number 50% (CC50).

#### HBV

Antiviral activity of R430 was determined by measuring the virus associated extracellular HBV DNA in HepG2.2.2.15 cells using real-time qPCR (TaqMan) as published^34^. Briefly, HepG2 2.2.15 cells were plated in 96-well plates at sub-confluent levels, followed by treatment in triplicates with serially diluted compounds next day and incubated at 37 °C with 5% CO_2_. At three days post-treatment, cell culture media was replenished with fresh compounds. Six days after the experiment initiation, cell culture supernatant was treated with pronase and used for the measurement of the virus associated DNA using real time qPCR, according to the standard protocol. Extracellular HBV DNA copy numbers were normalized to untreated controls to determine EC50 and EC90. Antiviral compound, Lamivudine (3TC), was used as a positive control in each assay. Cytotoxicity was measured by CellTiter® 96 Reagent, (Promega) uptake assay to determine CC50. Selectivity index 50% (SI50) were calculated by the ratio of CC50/EC50 and CC50/EC90.

#### HCV

Inhibition of HCV replication was measured in huh7 cells harboring HCV replicon (GT 1b-Con1 strain)-containing luciferase (luc-ubi-neo/ET) reporter gene according to the previously published protocol^35^. The luciferase reporter in this assay is an indirect measure of HCV replication, as HCV replication is proportional to the luciferase activity. Sub-confluent Luc-ubi-neo/ET cells plated in 96-well plates in triplicates were treated with various concentrations of the compounds and incubated at 37 °C with 5% CO_2_. Luciferase activity (as a measure of HCV replication) was measured 72-hours later, using Steady-Glo Luciferase assay reagent (Promega). Each run also included two positive controls-the NS5B inhibitor Sofosbuvir, and recombinant human interferon alpha-2b (rIFNα-2b). Cell viability was measured by CytoTox-ONE™ Homogeneous Membrane Integrity Assay (Promega) in parallel to determine CC50. Selectivity index 50% (SI50) were calculated by the ratio of CC50/EC50 and CC50/EC90 as mentioned above for HBV assay.

#### Zika virus (ZIKV)

Inihibition of ZIKV infection was assayed in hiPSC derived NPCs in triplicate. NPCs derived from hiPSC line 73-56010-02 sub-clone F were plated on a matrigel-coated 96-well culture dish and grown for 24 hours to reach 80–85% confluence. Cells were infected with ZIKV strain FSS-13025 (from Cambodia) or PE-243 (from Recife, Brazil) at an MOI of 1.0 and removed after 1 h. Fresh culture medium with either R430 (0.01–50 µM) or vehicle (DMSO) was added the cells incubated for 48 h. NPCs were fixed with 4% PFA, permeabilized with saponin, and stained with anti-flavivirus group antigen mouse monoclonal antibody (Millipore MAB10216), followed by goat anti-mouse polyclonal secondary antibody (LifeTech A10680 Alexafluor 488). Cells were counted on a BD Fortessa LSR flow cytometer and analyzed using FlowJo software. To estimate cytotoxicity, NPCs incubated in in mTESR medium in 96-well plates and incubated for 48 h with R430 or DMSO, after which they were harvested, stained with LIVE/DEAD Aqua (ThermoFisher L34966) and analyzed by flow cytometry with BD Fortessa LSR.

#### Analyses of ACV resistant HSV-1 strains

NPCs were grown to 85–90% confluence in matrigel-coated 96-well plates and infected with acyclovir-resistant HSV-1 strain tk- or PAAv at MOI 0.1. At 24 hours post-infection cells were fixed in 4% PFA, permeabilized with 0.2% Triton X-100, stained with antibody to ICP4 (Abcam ab6514) followed by alexa-488 anti-mouse secondary, and counterstained with Hoechst 33342. Cells were imaged with Nikon AS1 microscope and counted using Nikon NIS-elements software. IC50 values for ACV and R430 against mutant virus strains and KOS control strain were calculated using 4-parameter log logistic curve implemented using the DRC R package.

#### Explanted murine trigeminal ganglia with latent HSV-1 infection

The mouse explant co-cultivation model is a robust reactivation model in which latently infected trigeminal ganglia (TG) established by ocular infection are excised, dispersed and co-cultured with indicator cells as previously described^24^. ND4 Swiss mice (6–8 weeks old) were infected with light corneal scarification in both eyes using 1 × 105 plaque forming units, (pfu)/eye of HSV-1 strain 17+. At latency (28 days post-infection, dpi), TG were removed and individually placed in culture with indicator rabbit skin cells (RSC) in 24-well plates. Reactivation was followed over 21 days at 37 °C in the presence of either R430 (1 µM) or vehicle (DMSO). Culture media was changed every 2 days.

#### hiPSC based assays for HSV-1 infection

We used a genetically engineered HSV-1 KOS strain that expresses enhanced green fluorescent protein (EGFP) and red fluorescent protein (RFP) under the control of immediate early and late gene promoters, respectively^36^. To determine the optimal conditions for evaluating drug effects, hiPSC-N cells were infected for 2 hours (multiplicity of infection, MOI = 0.3), after which the inocula were replaced with media containing R430 (10 µM) or vehicle (DMSO). Separately, cells were incubated in media containing R430 (10 µM) or vehicle in the absence of HSV-1. All assays were conducted in triplicate. DNA was extracted from cells after 6 hours post infection (hpi), 12 hpi, or 24 hpi using (QIAamp DNA Mini Kit, (Qiagen) and the EGFP locus was amplified to obtain viral copy number using custom primers as previously described^37^. A significant increase in viral copy number was noted in vehicle treated cells after 12 h and 24 h, but not after 6 h (Supplementary Fig. 1). Substantial reduction in viral copy number was found after incubation with R430 at 12 h or 24 h. The 12 h incubation period was therefore selected for subsequent studies examining gene expression (see below).

RNA sequencing: At 12 hpi, the cellular RNA was extracted (RNeasy Mini Kits, Qiagen) and quantified using Agilent 4200 TapeStation (Agilent Technologies). Total RNA libraries were generated using the Illumina TruSeq Stranded Total RNA Sample Preparation Guide, Revision E. The first step involved the removal of ribosomal and mitochondrial RNA using biotinylated, target-specific oligos combined with Ribo-Zero rRNA removal beads. Following purification, remaining RNA was fragmented using divalent cations under elevated temperature, which were then copied into first strand cDNA using reverse transcriptase and random primers, followed by second strand cDNA synthesis using DNA Polymerase I and RNase H. Subsequently, a single adenosine base was added to each of the cDNA fragments, followed by ligation of an adapter. The products were purified and enriched with PCR to create the final cDNA library. The cDNA libraries were validated using KAPA Biosystems primer premix kit with Illumina-compatible DNA primers and Qubit 2.0 fluorimeter. Quality was examined using an Agilent Bioanalyzer Tapestation 2200. The cDNA libraries were pooled at a final concentration 1.8pM. Cluster generation and 75 bp paired-read dual-indexed sequencing was performed on Illumina NextSeq 500 (Children’s Hospital of Pittsburgh, University of Pittsburgh). Sequencing read quality was assessed using fastQC v0.11.4 and CLCbio v9.5.3 software. The average number of reads per sample was 102.6 million reads (51.3 million pairs, standard deviation, (SD) = 8.9 million reads). Ambiguous bases accounted for <0.05% of bases read and only 0.01% of reads had one or more ambiguous bases. Sequences were trimmed based on quality score using the modified-Mott trimming algorithm as implemented in CLC bio software, using a trim cutoff error probability of 0.05. Ambiguous bases were trimmed using a post trim maximal ambiguous base cutoff of 2. The trimmed reads were then mapped to the human genome GRCh38/hg38, using sequence and annotation provided by Ensembl (release 82). Approximately 93% of reads were mapped in pairs (SD = 0.45) across all samples, and 98.4% of reads were mapped in total (SD = 0.46). The remaining unmapped reads were collected and mapped to the Human Herpesvirus strain KOS genome (GenBank: JQ780693.1). The data were deposited in NCBI’s Gene Expression Omnibus database (https://www.ncbi.nlm.nih.gov/geo/query/acc.cgi?acc=GSE111656).

Quantitative RT-PCR (qRT-PCR): We used qRT-PCR to estimate gene expression for six genes using TaqMan assay probes (RHOB_Hs.00269660_s1, IRS1_Hs.00178563_m1, EIF4EBP1_Hs.00607050_m1, DDIT3_Hs.00358796_g1, DDIT4_Hs.01111686_g1 and EIF2AK3_Hs.00984003_m1). RNA samples used for RNA sequencing were subjected to cDNA synthesis with SuperScript III recommended protocol (Invitrogen). Next, qRT-PCR was performed in triplicate using the target and reference Applied Biosystems TaqMan probes specified by the manufacturer (ThermoFisher Scientific). Beta actin (ACTB) pre-designed Taqman probes were used as an endogenous control to normalize levels of the cDNA target and the comparative Ct method for quantification^38^. The ΔCt value for each target was determined by subtracting the average β-actin Ct value from the average target Ct value. The standard deviation of the difference was calculated from the standard deviations of the target gene and β-actin values. From ΔCt values, we calculated ΔΔCt values for fold changes by using the formula 2^(−ΔΔCt)^.

Chromatin immunoprecipitation (ChIP) analysis: hiPSC-neurons generated in matrigel-coated 6-well plates (1.5–2 × 10^6^ cells/well) were infected at Multiplicities of Infection (MOI) of 0.3. Two hours after the infection, cells were exposed to ACV (50 uM) or R430 (10 uM) for 24 hours. Chromatin was prepared from both conditions. Following chromatin cross-link and cell lysis, the chromatin was sonicated to generate fragment size of 100–500 bp. ChIP was performed using the ChromataChIP kit (Novus Biological) according to manufacturer’s instruction with 2 µ g of anti-histone H3K27me3 antibody (Millipore). Negative controls were samples to which no antibody was added (No Ab). Input DNA (non-immunoprecipitated) and immunoprecipitated DNA, and DNA from No Ab condition recovered from infected cultures exposed to ACV or R430 were analyzed by TaqMan real-time PCR in triplicate using primer pairs specific for the following HSV-1 genes: ICP4, ICP27, and LAT regions. Primer sequences: ICP4pF (5′-GACGTAGCACGGTAGGTCAC-3′); ICP4pR (5′-CTTTTTCCCACCCAAGCAT-3′); ICP4p probe (5′-6FAM-CCGTCGACGCGGAACTAGCG-TAMRA-3′); ICP27pF (5′-CGGCCTGACAGAGCTGTATT-3′); ICP27pR (5′-CCGAGAGGATGATGGAACAG3′); ICP27p probe (5′-6FAM-AAGGGGCTGTCGGGCGTC-TAMRA-3′); LATpF (5′-CAATAACAACCCCAACGGAAAGC-3′); LATpR (5′-TCCACTTCCCGTCCTTCCAT-3′); LATp probe (5′-6FAM-TCCCCTCGGTTGTTCC-TAMRA-3′).

The following qPCR conditions were used: 95 °C for 12 min followed by 40 cycles of 95 °C for 15 sec and 55 °C for 1 minute.

Data analysis: ChIP experiments were carried out with three biological samples for each condition. ChIP-qPCR data were performed in triplicate and normalized using the “Percent Input” method^39^. The Cts from 1% input were normalized to 100% by subtracting Log2 of 100 (6.64). Fold difference between immunoprecipitated (ChIP) samples and normalized input for each sample was calculated as follows: % Input = 2^[(Ct Input-6.64)-(Ct ChIP)]^ × 100. The ‘no-antibody’ signal was subtracted from the ChIP signal for each target gene. The enrichment of H3K27me3 at the promoter region of the indicated HSV-1 genes after the treatment with ACV or R430 was assessed using Student’s *t*-test.

Autophagy assay: Uninfected human TERT immortalized corneal epithelial cells that constitutively expresses the autophagy indicator protein LC3 fused to EGFP were incubated with R430. These cells indicate induction of autophagy by re-localization of LC3-GFP to puncta located in the cytoplasm in autophagosomes.

#### Statistical analysis

We estimated the EC50 and EC90 (compound concentration that reduces viral replication by 50% and 90%, respectively), CC50 (compound concentration that reduces cell viability by 50%) and SI90 (CC50/EC90) using R, package drc (version 3.0-1). The edgeR test was used to compare expression of individual transcripts between groups^40^.

Bioinformatics analyses: Functional analysis of differentially expressed genes (DEG) was performed using Qiagen’s Ingenuity Pathway Analysis (IPA, Qiagen Bioinformatics, https://www.qiagenbioinformatics.com/products/ingenuity-pathway-analysis/). IPA provides tools to interpret DEG datasets in the context of biological pathways^41^. Canonical pathway analysis identified biological pathways from the IPA library of canonical pathways that were most significant in relation to the R430-treated DEG data set. The significance of the association was measured by (1) a ratio (the number of genes from the data set mapped to the pathway divided by the total number of genes present in the pathway-map) and (2) a p-value, calculated by Fisher’s exact test. Pathways Activity Analysis, a function of IPA, enables prediction of the overall activation/inhibition states of the canonical pathways based on a z-score algorithm.

## Electronic supplementary material

Supplementary Information
