# Supplementary material for: R430: A potent inhibitor of DNA and RNA viruses
Source: Sci Rep. 2018 Nov 9;8:16662. doi: 10.1038/s41598-018-33904-y (PMC6226428; doi:10.1038/s41598-018-33904-y)
Supplement: Supplementary file 1 — Supplementary Information [file 41598_2018_33904_MOESM1_ESM.pdf]

R430: A potent inhibitor of DNA and RNA viruses

Leonardo D'Aiuto[1+], James McNulty[2+], Caroll Hartline[3], Matthew Demers[1], Raj Kalkeri[4], Joel Wood[1], Lora McClain[5], Ansuman Chattopadhyay[1], Yun Zhi[6], Jennifer Naciri[1], Adam Smith[1], Robert Yolken[7], Kodavali Chowdari[1], Carlos Zepeda-Velazquez[2], Chanti Babu Dokuburra[2], Ernesto Marques[8], Roger Ptak[4], Paul Kinchington[9], Simon Watkins[10], Mark Prichard[3], David Bloom[11], Vishwajit Nimgaonkar*[1, 12]

[1]Department of Psychiatry, Western Psychiatric Institute and Clinic, University of Pittsburgh School of Medicine, 3811 O'Hara Street, Pittsburgh, PA 15213

[2] Department of Chemistry and Chemical-Biology, McMaster University, 1280 Main Street West, Hamilton, Ontario, L8S 4M1 Canada

[3] University of Alabama at Birmingham, UAB School of Medicine, 1720 2nd Ave. S. Birmingham, AL 35294-3412

[4]Department of Infectious Disease Research, Drug Development, Southern Research Institute, 431 Aviation Way. Frederick, Maryland 21701

[5]Magee-Women's Research Institute, 204 Craft Ave, Pittsburgh, PA 15213

[6]Department of Pharmacology and Pharmaceutical Sciences, School of Medicine, Tsinghua University, Beijing, China

[7]Division of Neurovirology, Department of Pediatrics, Johns Hopkins University School of Medicine, 600 North Wolfe Street, Blalock 1105, Baltimore, MD 21287

[8] University of Pittsburgh, Department of Infectious Diseases and Microbiology, 9022 BST3, 3501 Fifth Avenue, Pittsburgh, PA 15260

[9]Department of Ophthalmology, University of Pittsburgh School of Medicine, 1016 Eye and Ear Institute, Pittsburgh, PA 15213

[10]Department of Cell Biology, University of Pittsburgh, 3500 Terrace Street, S362 Biomedical Science Tower (South), Pittsburgh, PA 15261

[11]Department of Molecular Genetics & Microbiology, University of Florida College of Medicine, Box 100266 JHMHC, Gainesville, FL 32610-0266

[12] Department of Human Genetics, Graduate School of Public Health, University of Pittsburgh, Pittsburgh PA 15213.

* vishwajitNL@upmc.edu

[+] these authors contributed equally to this work

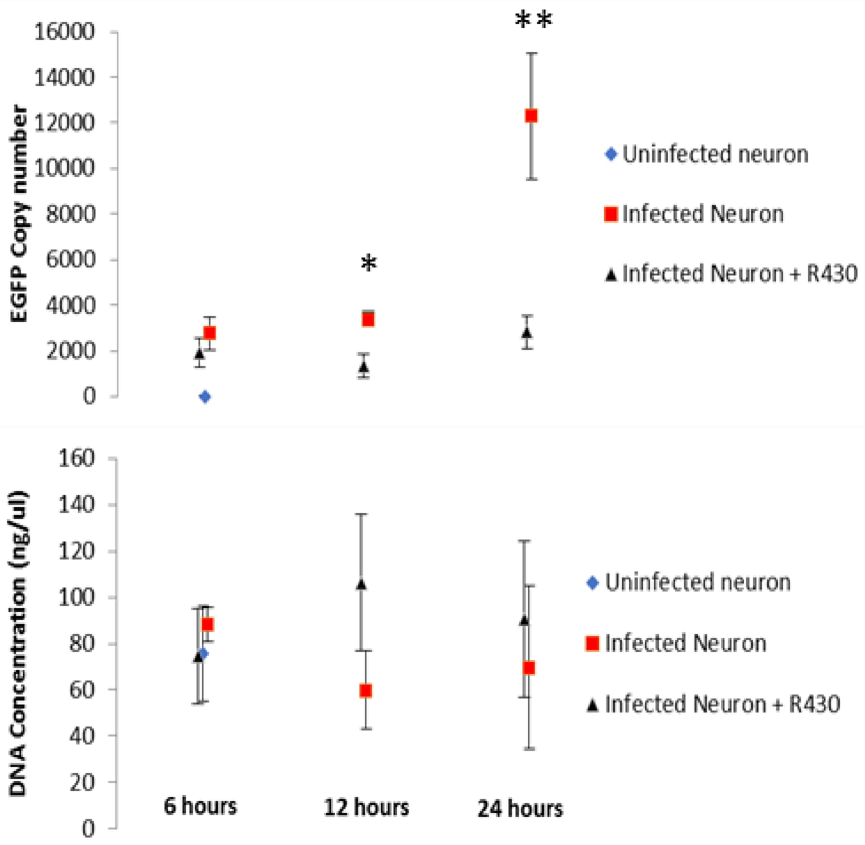

*:t=-5.38, p=0.004; **:t=-5.77, p=0.004

Supplementary Figure 1. Optimizing assays for HSV-1 infection. A genetically engineered HSV-1 KOS strain that expresses EGFP and RFP under the control of viral gene promoters was used to infect hiPSC-neurons cells were infected for 2 hours (multiplicity of infection, MOI=0.3), after which the inocula were replaced with media including R430 (10 µM) or vehicle (DMSO). Separately, cells were incubated in media with R430 (10 µM) or DMSO in the absence of HSV-1. All assays were conducted in triplicate. Cells were harvested at different time points as shown, DNA extracted and the EGFP locus was amplified to estimate viral copy number (upper panel). The total DNA concentrations are shown in the lower panel.

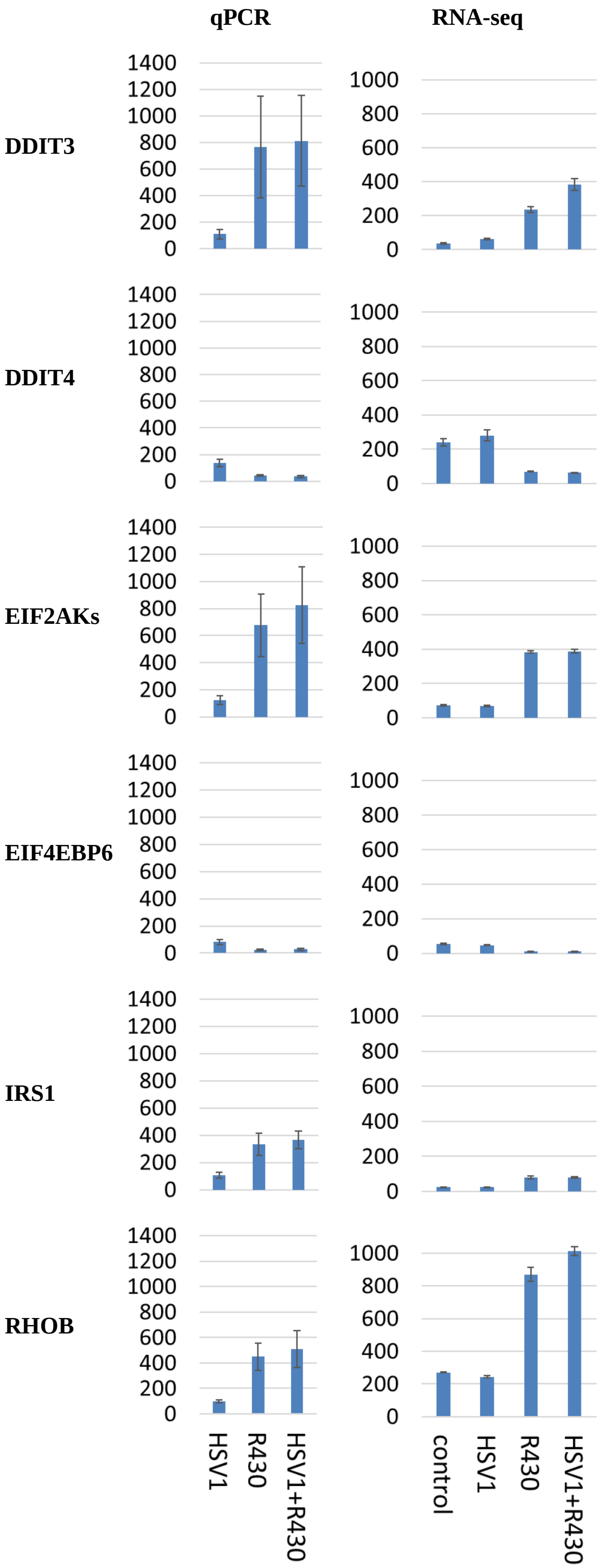

Supplementary Figure 2. Comparison of RTqPCR and RNA sequencing assays for selected human transcripts. Quantitative RT-PCR was used to estimate expression of six genes that showed significant R430-induced changes in transcript levels in the RNA sequencing experiments. Beta actin was used as an endogenous control. Left panel: Changes in ΔΔCt values in experimental conditions / uninfected control x 100; Right panel: TPM values for respective gene. Values shown as means and error bars denote standard deviations.

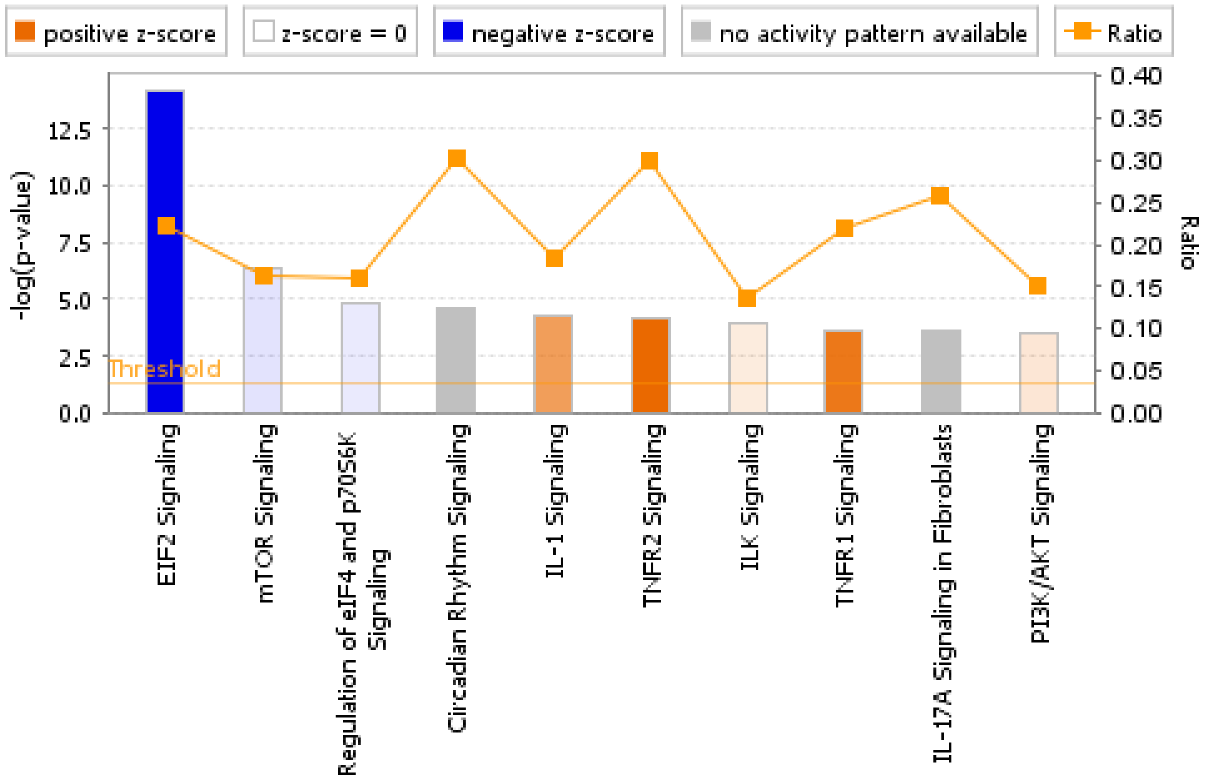

Supplementary Figure 3: Human gene pathways significantly altered by R430. Differentially Expressed Genes (DEG) measured by comparing R430 treated vs. vehicle-treated cells were subjected to Ingenuity Pathway Analysis (IPA). The top 10 pathways showing the greatest statistical significance (Fisher's Exact Test *p-value*) are shown. Activation (+ve z-score, orange bars) or inhibition (-ve z-score, blue bars) of each pathway is a measure of experimentally determined gene expression changes reported in the literature. The intensity of color indicates the degree of activation/inhibition. Pathways without an activity pattern are in grey. *Ratios* (orange square above each pathway) represent the degree of overlap between DEG and all members of a given pathway. Y-axis on the right indicates ratio values.

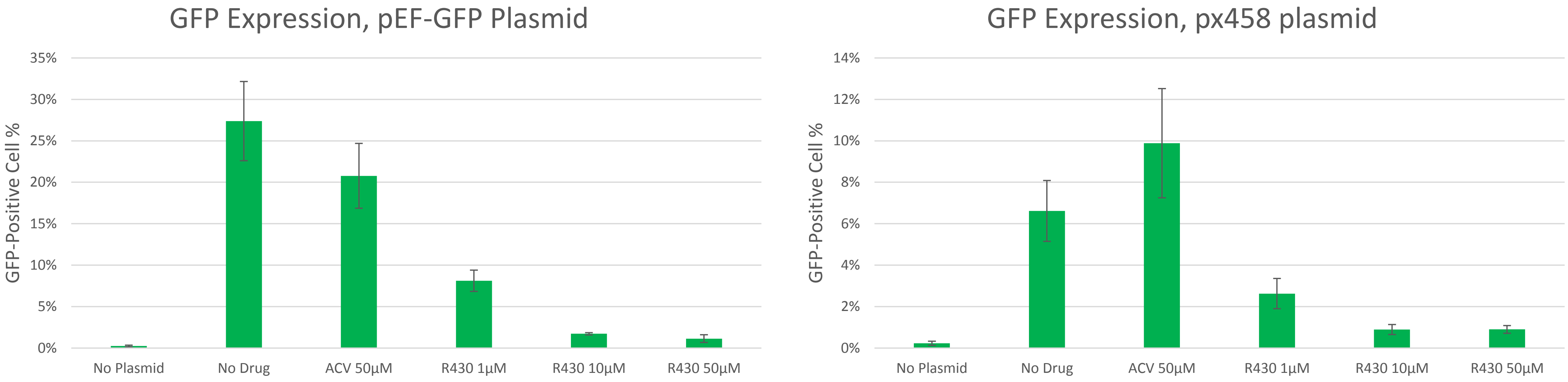

Supplementary Figure 4. R430 suppresses GFP expression in additional transient plasmid transfection assays. HEK293T cells were transfected with one of two plasmids, pER-GFP or px458. At 2 hours post-transfection. R430 (1, 10 or 50 µM), Acyclovir (ACV, 50 µM), or DMSO control was added (four replicates each). At 48 hours post-transfection, cells were evaluated for GFP expression by microscopy, then harvested and stained with Viability 780 dye (BioGems 62910-00), fixed and counted FACS. A general inhibitory effect on the expression of exogenous genes is plausible.

Supplementary Table 1

**Virus/ Strain**	**Cell line**	**Assay for antiviral efficacy/toxicity**	**Drug**	**EC50**	**CC50**	**SI50**
Zika / PE-243	NPC	Cells stained with flavivirus antibody/ LIVE/DEAD Aqua stain	R430	0.07	>50	>500
Zika / FSS-13025	NPC	Cells stained with flavivirus antibody/ LIVE/DEAD Aqua stain	R430	0.1	>50	>500
HSV-2/ G	HFF	CellTiter-Glo (Cytopathic effect & Toxicity)	R430	>0.24	0.28	<1
			Acyclovir	4.23	>150	>35
Human CMV / AD169	HFF	CellTiter-Glo (Cytopathic effect &Toxicity)	R430	>0.05	0.2	<4
			Ganciclovir	0.31	>150	>478
Murine CMV/Smith	HFF	qPCR(DNA)/CellTiter-Glo	R430	1.07	3.13	3
			Ganciclovir	0.08	>150	>1875
HBV / ayw1	HepG2 2.2.15	qPCR(DNA)/CellTiter-96	R430	0.45	0.62	1
			3TC	0.01	>2	>117
HCV / CON-1, Genotype 1b	Huh7	Luciferase reporter (Replicon)/CytoTox-1	R430	0.14	0.12	1
			PSI-7977	0.07	>5	>71
Zika / PE-243	NPC	Cells stained with flavivirus antibody/ LIVE/DEAD Aqua stain	R430	0.07	>50	>500
Zika / FSS-13025	NPC	Cells stained with flavivirus antibody/ LIVE/DEAD Aqua stain	R430	0.1	>50	>500
HSV-1 / *tk*-	NPC	Levels of viral ICP4 protein / cytopathic effects	R430	0.71	7.4	10
			ACV	44.7	>50	>1
HSV-1 / PAAv	NPC	Levels of viral ICP4 protein / cytopathic effects	R430	0.95	7.4	7.8
			ACV	>50	>50	>1

Supplementary Table S1. Potency and Toxicity of R430 against additional RNA and DNA viruses. The potency and toxicity of R430 was tested against additional RNA and DNA viruses as described in the Methods section. All values are in μM. ZIKA: Zika virus; HCV: Hepatitis C virus, HSV-2: Herpes Simplex virus, type 2; HCMV: Human cytomegalovirus; MCMV: murine cytomegalovirus; HBV: Hepatitis B virus. NPC: neural progenitor cells. HFF: human foreskin fibroblast. EC50: compound concentration that reduces viral replication by 50%, CC50: compound concentration that reduces cell viability by 50%; SI50: CC50/IC50.
